# “Lacking warmth”: Alexithymia trait is related to warm-specific thermal somatosensory processing

**DOI:** 10.1016/j.biopsycho.2017.07.012

**Published:** 2017-09

**Authors:** Khatereh Borhani, Elisabetta Làdavas, Aikaterini Fotopoulou, Patrick Haggard

**Affiliations:** a Institute of Cognitive Neuroscience, University College London, London, UK; b Department of Psychology, University of Bologna, Viale Berti Pichat 5, 40127 Bologna, Italy; c CSRNC, Centre for Studies and Research in Cognitive Neuroscience, University of Bologna, Viale Europa 980, 47521 Cesena, Italy; d Institute of Cognitive and Brain Sciences, Shahid Beheshti University, Tehran, Iran; e Division of Psychology and Language Sciences, University College London, London, UK

**Keywords:** Alexithymia, Somatosensory processig, Emotion processing, Quantitative sensory testing (QST), Thermal perception

## Abstract

Alexithymia is a personality trait involving deficits in emotional processing. The personality construct has been extensively validated, but the underlying neural and physiological systems remain controversial. One theory suggests that low-level somatosensory mechanisms act as somatic markers of emotion, underpinning cognitive and affective impairments in alexithymia. In two separate samples (total N = 100), we used an established Quantitative Sensory Testing (QST) battery to probe multiple neurophysiological submodalities of somatosensation, and investigated their associations with the widely-used Toronto Alexithymia Scale (TAS-20). Experiment one found reduced sensitivity to warmth in people with higher alexithymia scores, compared to individuals with lower scores, without deficits in other somatosensory submodalities. Experiment two replicated this result in a new group of participants using a full-sample correlation between threshold for warm detection and TAS-20 scores. We discuss the relations between low-level thermoceptive function and cognitive processing of emotion.

## Highlights

• Alexithymia scores were related to scores on a quantitative sensory testing battery.
• Two separate studies found altered low-level somatosensation in alexithymia.
• High alexithymic participants showed specific reduction in sensitivity to warm temperature.

## 1 Introduction

Alexithymia is a multifaceted personality construct, expressed with varying intensity in the general population. Self-report measures like the Toronto Alexithymia Scale (TAS) (Bagby, Parker, & Taylor, 1994), the most widely used and well-validated assessment tool (Bagby et al., 1994; Parker, Michael Bagby, Taylor, Endler, & Schmitz, 2003) characterise alexithymia through three main facets: difficulties in identifying feelings (DIF), difficulties in describing feelings (DDF), and externally-oriented thinking or a preoccupation with the details of external events (EOT). Dimensional analysis suggest alexithymia comprises an affective dimension, involving emotionalizing, and fantasizing, and a cognitive dimension, involving identifying, differentiating and describing feelings (Goerlich-Dobre, Bruce, Martens, Aleman, & Hooker, 2014; Herbert, Herbert, & Pollatos, 2011; Parker, Keefer, Taylor, & Bagby, 2008; Taylor, Bagby, & Parker, 1991). Importantly, people with high levels of alexithymia exhibit difficulties not only in processing their own emotions, but also in processing the emotions expressed by others (Borhani, Borgomaneri, Làdavas, & Bertini, 2016; Jessimer & Markham, 1997; Parker et al., 1993; Parker, Prkachin, & Prkachin, 2005; Scarpazza, Di Pellegrino, & Làdavas, 2014; Sifneos, 1973). Thus, alexithymic individuals show altered recognition of emotional stimuli (Grynberg et al., 2012, Ihme et al., 2014) and decreased activation of the amygdala during presentation of emotional stimuli (Jongen et al., 2014; Moriguchi & Komaki, 2013), and particularly negative stimuli (Kugel et al., 2008; Pouga, Berthoz, de Gelder, & Grezes, 2010; Reker et al., 2010; for a recent metaanalysis: van der Velde et al., 2013).

The relation between alexithymic traits and specific neural systems or pathways remains controversial. The multiple somatosensory pathways that transmit interoceptive, proprioceptive, mechanoreceptive and nociceptive information to the CNS are a plausible candidate. The view of emotions as linked to bodily states, or ‘somatic markers’ has long been popular (Damasio., 1999; Damasio, Everitt, & Bishop, 1996; James, 1890). Indeed, emotion-specific responses have been reported in primary somatosensory cortex (Sel, Forster, & Calvo-Merino, 2014). Such theories suggest that alexithymia might involve abnormalities of somatosensory or autonomic processing. Disruption in regulation of emotions in alexithymia, particularly negative emotions, is thought to result in chronic sympathetic hyperarousal, high sensitivity to painful stimulation, somatosensory amplification (the tendency to experience mild somatic sensations as intense, noxious, and disturbing – Barsky, Goodson, Lane, & Cleary, 1988) and complaints of physical symptoms (Kano, Hamaguchi, Itoh, Yanai, & Fukudo, 2007; Lumley, Stettner, & Wehmer, 1996). Brain regions previously associated with somatosensory processing, including the insula and somatosensory cortices (Dijkerman & De Haan, 2007; Iwamura, 1998) were hypothesised to be overactive during emotional processing in alexithymia, potentially explaining the tendency to experience physical symptoms when emotionally aroused (Karlsson, Näätänen, & Stenman, 2008). However other studies failed to find associations between somatosensory amplification and alexithymia (Gregory, Manring, & Berry, 2000; Kosturek, Gregory, Sousou, & Trief, 1998; Lesser, Koehle, & Lueders, 1979). Moreover, the frequent comorbidity of alexithymia with several psychiatric disorders may contribute to such discrepancies. For instance, while Nyklíček and Vingerhoets (2000) reported hypersensitivity for pain and touch in alexithymia, de Zwaan, Biener, Bach, Wiesnagrotzki, and Stacher (1996) failed to find any association between the degree of alexithymia and thermally and mechanically induced pain thresholds, in a group of eating disorder patients with alexithymia (de Zwaan et al., 1996). Table 1.

**Table 1:** Summarizes a number of key studies addressing somatosensory processing in alexithymia.

*Studies* relating alexithymia to atypical somatosensory processing					
No	Authors and year	*N*	Participants	Experimental method	Results
1.	Wise et al. (1994)	101	Psychiatric patients	Self reports: TAS-20 Somatosensory Amplification Scale (SSAS)	Significant positive correlation between TAS-20 and SSAS scores
2	Nyklíček and Vingerhoets (2000)	41	Healthy participants	Painful electrical stimulation	Significant positive correlation between alexithymia score and sensitivity to pain
3	Nakao et al. (2002)	81	Psychosomatic vs patient control groups	Self reports: TAS-20 SSAS	Significant positive correlation between TAS-20 and SSAS scores
4	Kano et al. (2007)	45	Healthy participants	Brain processing of visceral sensation induced by colonic distension	Significant positive correlation between alexithymia and sensitivity to visceral stimulation
5	Millard and Kinsler (1992)	195	Patients with chronic nonmalignant pain	Self report: TAS Pain intensity	No relation between alexithymia and sensitivity to pain
6	Cox et al. (1994)	55	Patients with somatoform pain disorder	Self report: TAS_20 McGill Pain Questionnaire	No significant pain severity difference between alexithymics and non-alexithymics
7	de Zwaan et al. (1996)	72	Patients with anorexia nervosa, Patients with bulimia nervosa, Healthy participants	Detection threshold for mechanically and thermally induced pain	No relation between alexithymia and pain threshold
8	Gregory et al. (2000)	140	Patients with chronic nonmalignant pain	Self reports: TAS SSAS	No significant association between alexithymia and SSAS score
9	Jackson et al. (2002)	116	Healthy participants	Pain tolerance to cold pressor test	No significant correlation between alexithymia and sensitivity to unpleasant stimuli
10	Karlsson et al. (2008)	21	Healthy participants	Watching emotional videos	Somatosensory brain regions were more activated during watching emotional videos in HA than LA

This summary shows a high heterogeneity of both methods and conclusions in previous research. Indeed, existing accounts make conflicting predictions about the *direction* of any putative association between alexithymia and somatosensation. On the one hand, somatosensory cortical areas (e.g. right primary and secondary somatosensory areas and the insula) are positively activated during emotion recognition in healthy volunteers (Adolphs, 2002), suggesting that high alexithymia might be associated with *reduced* somatosensory processing. On the other hand, the somatosensory amplification concept predicts increased somatosensory processing at least for some somatosensory modalities such as pain, and visceral sensation (Barsky et al., 1988, Ihme et al., 2014; Kano et al., 2007; Perez, Barsky, Vago, Baslet, & Silbersweig, 2015).

Detailed understanding of relations between alexithymia and somatosensory processing has been hampered by the lack of psychophysiologically-validated measures of somatosensory processing in the alexithymia literature. Somatosensory systems can be divided into several submodalities, each reflecting a distinct class of stimulus energy, peripheral receptor, afferent pathways and cortical target. Within thermoception, for example, warm and cold sensations are mediated by different afferent fibres, and are therefore considered distinct submodalities. Some somatosensory submodalities, such as mechanoreceptive light touch, are classically interpreted as exteroceptive, while others, such as visceral baroreception, are considered interoceptive. The theoretical and empirical confusion over somatosensory processing in alexithymia may arise partly from lack of a systematic experimental approach to measuring somatosensation itself. Few studies of alexithymia have taken account of modern psychophysiological approaches to pathway-specific somatosensory perception, and few have tested *multiple* somatosensory submodalities. Thus, the existence and specificity of any link between somatosensory processing and alexithymia remains unclear.

We therefore performed two studies relating alexithymia scores to performance on established psychophysiological quantitative sensory testing (QST) battery (Rolke, Baron et al., 2006). QST involves multiple psychophysical tests, assessing the neurophysiological function of the major somatosensory afferent fibre pathways from the skin (Hansson, Backonja, & Bouhassira, 2007). QST is widely used in neurology, and extensive normative data are available (Rolke, Baron et al., 2006, Rolke, Magerl et al., 2006). Importantly, each QST subtest focuses on a specific somatosensory submodality, so QST has potentially high sensitivity to identify selective deficits in specific neurophysiological pathways. We used the following QST subtests: warm threshold, cold threshold, pinprick radiant heat pain threshold, and somatosensory detection. These tests are thought to involve the following afferent pathways respectively: somatosensory detection – Aβ primarily, but potentially also Aδ and C-fibres; warm – C-fibre; cold – Aδ-fibre – pinprick radiant heat pain- Aδ fibres. In the latter case, our methods (see later) set the stimulus intensity above the Aδ threshold, which reliably produces a distinctive pinprick sensation. However, there is inevitably a concurrent activation of lower-threshold C-fibres signaling innocuous warmth.

In addition, we included established tests of tactile acuity (Aβ-fibre), interoceptive awareness (thought to depend on mechanosensitive receptors innervated by vagal afferent fibres in the atria and venoatrial junction, or mechanosensitive C-fibres in the ventricles (Longhust, 2004; Malliani, Lombardi, & Pagani, 1986), by intra-thoracic detection of the force generated by changing blood-pressure on the walls of the great vessels in surrounding mechanosensitive thoracic tissues (Eichler and Katkin, 1994; ; Schandry et al., 1993), and possibly also by cutaneous mechanosensitive fibres overlying large arteries (Critchley, Wiens, Rotshtein, Öhman, & Dolan, 2004; Khalsa, Rudrauf, Sandesara, Olshansky, & Tranel, 2009)), and affective touch (presumed C-tactile mechanoreceptor pathway McGlone, Wessberg, & Olausson, 2014, though concurrent activation of Aβ cannot be avoided). These tests are not part of classical QST, but involve a degree of psychophysiological specificity, and which have previously been linked to alexithymia. We hypothesised that if emotion recognition impairment in alexithymia has a somatosensory grounding, this should be revealed by an association between TAS scores and performance on one or more subtests of QST. Based on previous results, we predicted that any such association would involve the ‘protopathic’, small-fibre submodalities that participate in conveying somatic states, rather than the ‘epicritic’, large-fibre submodalities that participate in somatosensory exteroception.

## 2 Experiment 1

### 2.1 Methods

#### 2.1.1 Participants

In the absence of previous similar studies, we were interested in whether a strong association between somatosensory processing and alexithymia existed, so we based our a priori power calculation on a relatively large assumed effect size (0.90). A power calculation in G*Power 3.1 (Faul, Erdfelder, Lang, & Buchner, 2007) indicated that 40 participants would achieve a power of approximately 0.80 in a two-tailed between-groups independent *t*-test.

189 volunteers (fifty one males, mean age = 23.7, range:18–40) filled out the 20-item Toronto Alexithymia Scale (TAS-20; Taylor, Bagby, & Parker, 2003) online. TAS scores range from 20 to 100. Scores of 61 or higher were considered as high alexithymia, while scores of 36 or lower were considered as low alexithymia (Taylor, Ryan, & Bagby, 1985). For experiment 1, 20 healthy Individuals with high TAS-20 totals (six males, mean age = 24.65, range: 19–35) and 20 with low TAS-20 totals (six males, mean age = 25.7, range:19–40) TAS-20 totals were selected, using the cutoffs defined above, in order to obtain an experimental sample varying widely in levels of the alexithymia trait. The 20 low alexithymia (LA) participants had a TAS mean score of 31.05 (SD = 3.18, range: 25–35, corresponding to the lower TAS quartile reported in the general population (Taylor et al., 1985), while the 20 high alexithymia (HA) participants had a TAS mean score of 66.2 (SD = 5.69, range: 61–77, corresponding to the upper TAS quartile reported in the general population). In addition, the alexithymia module of the structured interview for the Diagnostic Criteria for Psychosomatic Research (DCPR) (Mangelli, Semprini, Sirri, Fava, & Sonino, 2006; Porcelli & Rafanelli, 2010; Porcelli & Sonino, 2007) was also used to confirm the presence or absence of alexithymia. At least 3 of 6 characteristics in this interview must be present for alexithymia (Porcelli & Rafanelli, 2010). (LA:1.6, SD = 0.5, range:1–2;, HA:4.6, SD = 1.14, range:3–6). Participants were included in the study if i) they had no history of neurological, major medical or psychiatric disorder and ii) their scores on the TAS-20 and the DCPR were congruent (congruency was defined as both scores on TAS20 and DCPR had to indicate the same level of alexithymia. For instance, to be considered in high alexithymia group the score in TAS-20 had to be 61 or higher, and DCPR score had to be 3 or higher). No participant was excluded due to discrepancy between TAS-20 and DCPR score. Two participants were excluded after completing the TAS online, but prior to experimental testing, due to self-reported previous history of neurological or psychiatric disorder.

Experimental data were obtained from the 40 included healthy participants in a comprehensive standardized QST protocol consisting of 7 tests: cold and warm thermal perception thresholds, pinprick pain threshold, tactile acuity, affective touch, interceptive awareness, and somatosensory signal detection. All participants performed the tests in the same, stated order. Fixed order testing was necessary for technical reasons, and to avoid possible interactions between specific modalities and those tested subsequently (Gröne et al., 2012). For instance, it has been found that applying thermal stimulation before mechanical stimuli might lead to mechanical pain sensitivity (Gröne et al., 2012). The whole experiment took about two hours and a half. Participants provided written informed consent prior to the experiment and were paid £7.50 per h. The experiment was approved by the UCL Research Ethics Committee, and carried out in accordance with the provisions of the Declaration of Helsinki.

##### 2.1.1.1 Thermal detection threshold test

Contact thermal stimuli were delivered to the back of the left hand using a 13 mm circular diameter Peltier-type thermode (NTE-2A, Physitemp Instruments Inc). Contact warm and cold threshold was estimated by the method of limits (Yarnitsky, Sprecher, Zaslansky, & Hemli, 1995). The probe temperature was fixed for a random time between 28 and 30 s, an initial level of 32 °C, and then ramped up or down by 2 °C/s. To avoid possible pain and tissue damage, maximum temperature was limited to 50 °C and minimum temperature was limited to 14 °C. Participants were asked to press a button using their right hand as soon as they felt any change in temperature, and then report the direction of the change, whether the stimulus got colder or warmer. Three cold and three warm stimulus ramps were delivered in random order. The three trials were averaged to obtain a warm threshold and a cold threshold measurement. Interstimulus intervals were 20 s.

The fibre classes investigated by these tests are different. The warm threshold test was designed to investigate the small-diameter C fibres associated with innocuous warm perception, while the cold threshold test was designed to investigate the larger-diameter Aδ-fibres associated with cold perception.

##### 2.1.1.2 Pinprick pain threshold and discrimination task

Noxious radiant heat stimulation was delivered by an infrared CO2 laser stimulation device with a wavelength of 10.6 μm (SIFEC, Ferrières, Belgium). The laser pulse (100 ms duration) was transmitted via an optic fibre to reach a spot diameter of 6 mm on the dorsum of participants’ left hand. These laser pulses selectively excite Aδ- and small C-fibres, but do not co-activate the Aβ-fibres associated with mechanoreceptors. For each participant, we identified the Aδ threshold for ‘pinprick pain’ using ascending-descending-ascending staircases. The threshold was identified by finding the lowest skin temperature that elicited both a report of “pinprick sensation”, and a reaction time (RT) < 650 ms (Churyukanov, Plaghki, Legrain, & Mouraux, 2012; Mouraux, Guerit, & Plaghki, 2003). Starting at 38 °C, the temperature was increased in steps of 4 °C until RT was less than 650 ms. Then the temperature was decreased in steps of 2 °C until the RT became longer than 650 ms. Finally, the temperature was increased in steps of 1 °C until RT was less than 650 ms again, and the participant reported a pinprick sensation for 3 consecutive repetitions of the same temperature. To estimate nociceptive discrimination, we then set a low stimulus intensity at 2 °C above pinprick threshold, and the high stimulus intensity at 8 °C above pinprick threshold. Participants were familiarized with the high and low levels of stimulation, and received some discrimination practice trials. Then they performed a forced-choice task in which they received 30 low and 30 high intensity stimuli, randomly interleaved with a random 10–15 s interstimulus interval. After each set of fifteen stimuli there was a 5 min pause to prevent habituation to painful stimuli. The participants were required to identify pinprick pain as ‘high’ or ‘low’. Their reports in the task were recast as attempts to detect the high intensity stimuli, so that the results could be analysed using signal detection analysis (Green & Swets, 1966; Macmillan & Creelman, 1991). This analysis gave independent estimates of nociceptive perceptual sensitivity and response bias. We considered the number of hits for high intensity level of noxious or electro-tactile stimulus (number of high intensity stimulus trials in which participants responded ‘high’), false alarms (number of low intensity stimulus trials in which participants responded ‘high’). Hit rates [P(‘high’ response | high intensity stimulus), proportion of hit trials to which subject responded ‘high’] and false alarm rates [P(‘high’ response | low intensity stimulus), proportion of trials in which low intensity stimuli were reported as ‘high’] were calculated. These were used to obtain the perceptual sensitivity (*d*’) in detecting the high intensity stimulus. *D*’ does not require homogeneous variance and can be calculated using a standard correction and adjustment even if the hit or false alarm rates are 1 or 0) (Macmillan & Creelman, 1991; Stanislaw & Todorov, 1999). The tendency to report stimuli as ‘high’, irrespective of actual intensity, (C, response bias) was also obtained. Sensitivity and response bias were calculated for high intensity noxious and electro-tactile stimuli.

##### 2.1.1.3 Tactile acuity test

The Grating Orientation Test (GOT; Van Boven & Johnson, 1994) consists of a series of square wave gratings with graded spatial frequencies, and 50% duty cycle. It was used to measure each participant’s grating orientation discrimination threshold – an established test that probes light touch receptors and the associated large-diameter Aβ fibres. Beginning with the largest ridge width (3 mm) the experimenter applied the grating to the participant’s index fingertip while they were blindfolded. Each grating was presented three times for approximately 0.5 s, randomly changing the orientation, so the ridges could run either along or across the axis of the index finger. Participants made unspeeded verbal forced-choice judgments regarding the orientation of the gratings, responding “along” or “across” the finger. If all three trials were perceived correctly, the next lowest ridge width was used. This procedure continued with gratings of decreasing ridge width until the participant made at least one error (i.e., accuracy of 66.6% or less over three trials). The ridge width was then increased again until the participant answered correctly on 100% of three trials. This ridge width was then used as the participant’s threshold. The whole procedure repeated 3 times and results were averaged to obtain a single threshold score.

##### 2.1.1.4 Affective touch (putative C tactile system)

Participants sat at a table with their left forearm resting palm-up. Three tactile stroking stimuli at velocities of 0.3, 3, 30 cm/s were delivered over a 10 cm distance. The stimuli were delivered either by experimenter’s index, middle and ring finger or by three joined paintbrushes (Daler Rowney Oval Wash Brush size 1/2) positioned to form the same shape as the experimenters’ fingers, and moved by a robot (Phantom premium 1.0). Stimuli were blocked across the type of agent (experimenter or robot). Inside each block, stimulus speed was randomised. Each speed repeated twice. The interstimulus interval was 30 s to minimize receptor fatigue. To keep stimulation duration constant, 1 stroke at 0.3 cm/s, or 10 consecutive strokes at 3 cm/s or 100 strokes at 30 cm/s was applied, as in previous studies (e.g. Gentsch, Panagiotopoulou, & Fotopoulou, 2015; Löken, Wessberg, McGlone, & Olausson, 2009). The experimenter was trained to apply stroking similarly to the robot. Following each stroke the participants were instructed to rate the pleasantness and softness of stimulus using two separately-presented paper and pencil visual analog scales (VAS), with the endpoints unpleasant to pleasant (− 10 to 10). Prior to the experiment participants were familiarized with one trial for each stimulus with different velocity and delivered by either the experimenter or robot. Previous studies have shown an “inverted U-shaped” relationship between brushing velocity and firing rate in C-tactile afferents, with highest responses to 3 cm/s stroking. Further, this velocity range was also perceived as more pleasant than faster or slower stroking, suggesting an affective role for these afferents (Bessou, Burgess, Perl, & Taylor, 1971; Löken et al., 2009). Faster (30 cm/s) and slower (0.3 cm/s) velocities were included were used to control for arousal and number of strokes associated with C-tactile suboptimal velocities versus the optimal velocity. Importantly, therefore, any potential differences in affective touch processing between groups should show an interaction between group and stroking velocity, with the prominent difference between groups being at 3 cm/s.

This test is thought to probe a specific neural pathway for affective touch system which is mediated by small unmyelinated C-Tactile mechanoreceptors (CT) – though concurrent activation of Aβ mechanoreceptors cannot be avoided.

##### 2.1.1.5 Interoceptive sensitivity

The Heartbeat Perception Task was used as a measure of interoceptive sensitivity (ISt).The ECG was measured through nonpolarizable Ag-AgCl electrodes attached to the left ulna styloid process, and right wrist and referenced to the right radial styloid process. Signals were recorded by a BioSemi amplifier system (BioSemi, Amsterdam, The Netherlands). A sampling rate of 1000 Hz was used. R-waves were detected online and were stored on a trigger channel. The heartbeat perception task was performed according to the Mental Tracking Method proposed by Schandry (1981), using three intervals of 25, 45, and 60 s. The three perception intervals were separated by standard resting periods (30 s). Participants sat on a chair and were asked to close their eyes and silently count their heartbeats by concentrating on their heart activity. During heartbeat counting, participants were not permitted to take their pulse or to attempt any other physical manipulations that could facilitate the detection of heartbeats. Following the stop signal, participants were asked to verbally report the number of counted heartbeats. The participants were not informed about the lengths of the counting phases or about the quality of their performance. ISt was measured as a heartbeat perception score, calculated by taking the mean score across the three heartbeat perception intervals according to the following transformation: 1/3 Σ(1-(|recorded heartbeats – counted heartbeats|)/recorded heartbeats). The heartbeat perception score varies between 0 and 1. The maximum score of 1 indicates absolute accuracy of heartbeat perception. This heartbeat detection task is widely used to assess interoceptive sensitivity (Dunn, Dalgleish, Ogilvie, & Lawrence, 2007; Herbert, Pollatos, & Schandry, 2007). It has good test-retest reliability, and correlates highly with other heartbeat detection tasks (Knoll and Hodapp, 1992). Previous studies provided contrary results regarding the relation between ISt and alexithymia. While some studies reported a negative correlation between ISt and alexithymic trait (Herbert et al., 2011; Shah, Hall, Catmur, & Bird, 2016), another showed a positive correlation (Ernst et al., 2013)

##### 2.1.1.6 Somatosensory detection test

To assess the somatosensory detection threshold electrocutaneous stimulation was used. This potentially activates all the fibres having receptors in the stimulated skin region (large Aβ fibres subserving touch, Aδ fibres subserving fast pain, and small-diameter unmyelinated fibres subserving temperature). At the detection threshold intensities used here, however, nociceptive fibres were unlikely to be activated, and Aβ fibres probably contribute most to perceptual performance. Tactile stimulation with a duration of 10 ms was delivered using a Digitimer DS5 constant current stimulator (Digitimer Ltd., Welwyn Garden City, UK) connected to a pair of disposable press-stud electrodes (Biosense Medical, Chelmsford, UK) placed on dorsum of the left hand.

Electro-tactile stimulation was used to determine each participant’s detection threshold. Starting at 0.5 mA, the current was increased in steps of 0.5 mA until the participant detected the stimulus. The current was then reduced in 0.5 mA steps until the stimulus was no longer detected, and then increased again until the stimulus was again perceived. This last value was taken as the detection threshold. Next, the current was increased rapidly to near-pain threshold, and then the same procedure was used to measure the participant’s pain threshold. The low and high levels of stimulation for the main experiment were then set to 45% and 55%, respectively, of the range between the detection and pain thresholds. These levels were chosen based on a pilot study in a separate group of volunteers which indicated that this difference between high and low electro-tactile intensities would approximately match the discrimination performance used in the test of nociceptive discrimination between high and low laser heat-pain stimuli. The mean difference between the high and low intensities was 1.05 mA (range = 0.55–1.15 mA). Participants were familiarized with the high and low levels of stimulation. Then they performed a forced-choice task in which they received 80 (40 high stimulus intensity) randomly delivered stimuli. Inter stimuli interval was randomised between 8–10 s. Participants were asked to discriminate whether the perceived stimulus was high or low stimulus intensity, and respond with the keyboard. SDT was used to obtain independent estimates of perceptual sensitivity and response bias as described for pinprick radiant heat pain stimulus.

### 2.2 Results

**Fig. 1:**
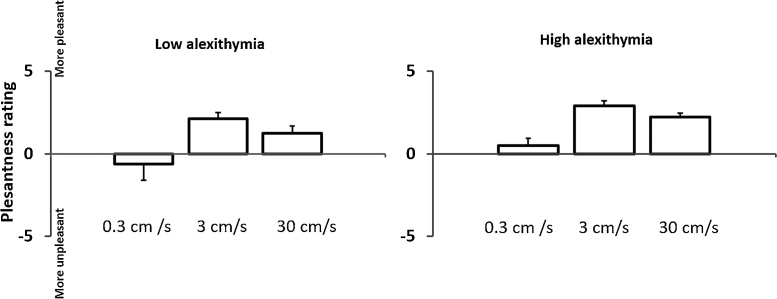
Affective touch: Mean pleasantness ratings for the three different stroking velocities in low and high alexithymia groups. Stroking at 3 cm/s was rated as significantly more pleasant than stroking at 0.3 or 30 cm/s. The High alexithymia group gave significantly higher ratings overall, but the interaction between group and stroking velocity was not significant.

We found no significant differences between the high alexithymia and low alexithymia groups in cold detection, pinprick radiant heat pain threshold, somatosensory detection, tactile acuity detection, affective touch (Fig. 1), (see Supplementary Tables S1a, S1b, S2a, S2b, and Fig. S1), and interoceptive sensitivity. However, the test of warm detection thresholds showed significantly higher warm thresholds in the high alexithymia group than in the low alexithymia group. The full results are shown in Table 2.

**Table 2:** QST and interoceptive sensitivity results.

Tested modality	High Alexithymia group		Low Alexithymia group		t-value	df	p-value	Effect size (Cohen’s d)
	Mean	SD	Mean	SD				
Warm threshold (°C)	36.73	3	34.43	1.46	3.08	38	0.004	0.97
Cold threshold (°C)	28.56	5.24	29.96	0.76	−1.18	38	0.24	−0.37
Warm detection accuracy (%)	80.11	40.80	80.11	40.80	0.00	38	1.00	0.00
Cold detection accuracy (%)	85.04	36.51	95.03	22.21	−1.04	38	0.30	−0.34

Pinprick laser heat-pain threshold (°C)	47.55	2.64	48.10	2.22	−0.71	38	0.48	−0.22
RTs to noxious laser stimulus (ms)	512	80.23	518.35	65.27	−0.27	38	0.8	−0.09
Sensitivity (*d’*)	1.79	0.75	1.82	0.56	0.12	38	0.9	−0.04
Response bias (C)	0.39	0.44	0.27	0.36	0.94	38	0.35	0.29

Tactile acuity threshold (mm)	1.66	0.38	1.61	0.36	0.42	38	0.67	0.13

Sensory detection task:								
Tactile detection threshold (mA)	1.50	0.56	1.58	0.73	−0.46	38	0.64	−0.12
Pain threshold (mA)	1.63	0.63	1.73	0.73	−0.48	38	0.63	−0.15
Sensitivity (*d’*)	1.40	0.49	1.69	0.62	−1.66	38	0.10	−0.51
Response bias (C)	0.08	0.28	−0.02	0.23	1.23	38	0.22	0.35

Interoceptive sensitivity	0.74	0.15	0.73	0.19	0.09	38	0.92	0.05

## 3 Experiment 2

Experiment 2 aimed to confirm the apparent selective increase in warm detection threshold in high alexithymics, using a new group of participants. This experiment focused only on warm and cold thermal threshold measures, since these provide a clear contrast between two independent somatosensory submodalities within a single dimension of sensory experience, i.e., thermoception. Further, instead of selectively picking extremes of the alexithymia distribution, we sampled more evenly across the population range of alexithymia scores in the general population, and used a regression analysis approach. This approach should identify whether a general relation exists between thermal perception thresholds and alexithymia traits.

### 3.1 Methods

#### 3.1.1 Participants

211 new volunteers filled out the TAS-20 online. From these respondents, we selected twenty healthy individuals with low, 20 with medium, and 20 with high TAS-20 total scores (n = 20, bottom tertile score 36≤, n = 20, middle Tertile score 36≤, 61≥, n = 20, top tertile score 61 ≥ ) to take part in thermal detection threshold experimental testing. The sampling strategy was based on the power calculation for the first experiment, with a modification to sample from all the tertiles of the TAS score, to coverage of the full range of alexithymia expression. However, in contrast to experiment 1, our statistical analysis plan used a regression model to investigate a possible continuous relation between thermoception and alexithymia. The thermal detection test was conducted as in experiment 1.

### 3.2 Results

A linear regression was calculated to predict warm detection threshold based on TAS-20 score across all 60 participants. A significant relation was found (F(1,58) = 15.14, p < 0.0001), *R^2^* = 0.207. No significant relation was found between cold detection threshold and TAS-20 score (F(1,58) = 0.664, p = 0.419), with *R^2^* of 0.011.

In further, exploratory analyses, we investigated relations between alexithymia subscales (Difficulty Identifying Feelings (DIF), Difficulty Describing Feelings (DDF), and Externally Oriented Thinking (EOT)) and warm detection thresholds. A linear regression was calculated to predict warm detection threshold based on DIF subscale. An adjusted significance level of 0.0133 was used, since 3 separate tests were performed, but the probabilities are reported uncorrected. A significant regression was found (F(1,58) = 9.92, p = 0.003), with an *R^2^* of 0.146. Likewise a significant regression was found between DDF subscale and warm threshold (F(1,58) = 17.35, p < 0.0001), with an *R^2^* of 0.230. No relation was found between EOT subscale and warm threshold (F(1,58) = 3.53, p = 0.065) with an *R^2^* of 0.057.

A further linear regression was calculated to predict accuracy in detecting direction of temperature change in the warm and cold threshold tasks. Percentage accuracy in detecting the direction of change was calculated for warm and cold stimuli separately. No significant relation was found between accuracy in detecting warm changes and TAS scores (F(1,58) = 0.012, p = 0.91), with an *R^2^* of 0.00, nor between accuracy in detecting cold changes and TAS scores (F(1,58) = 0.007, p = 0.93), with an *R^2^* of 0.00. This suggests there was no strong group difference in bias to respond ‘warm’ or ‘cold’.

Finally, a regression was performed to predict accuracy in detecting warm and cold temperature based on warm and cold detection threshold. No significant relation was found, neither for warm (F(1,58) = 0.42, p = 0.52) with an *R^2^* of 0.007, nor for cold temperature changes (F(1,58) = 2.05, p = 0.15) with an *R^2^* of 0.034. This suggests that the increase warm threshold in participants with greater TAS-20 scores is not simply due to differences in detection accuracy.

## 4 Discussion

Alexithymia is defined by difficulties in identifying and describing feelings, and a tendency to focus on external events rather than inner experiences (Taylor et al., 1991). Alexithymia has been characterised as a difficulty in cognitively mapping feeling states onto internal bodily responses (Taylor, 2000). Besides, alexithymia has been shown to be associated with atypicalities in sensory processing. While some studies have found hypersensitivity to some somatosensory modalities (e.g. pain, touch, heat, and visceral stimulation) in high alexithymics (Katz, Martin, Pagé, & Calleri, 2009; Kano et al., 2007; Kosturek et al., 1998; Nyklíček & Vingerhoets, 2000) others did not (Cox, Kuch, Parker, Shulman, & Evans, 1994; de Zwaan et al., 1996; Millard & Kinsler 1992 – see Table 1 for summary). Further, previous studies have used a wide variety of somatosensory tests, but studies applying a systematically-motivated set of validated tests of different somatosensory functions are rare. Therefore, it seems possible that emotion recognition difficulties in alexithymia could be caused by atypical somatosensory processing.

We have investigated this issue using QST as an established, standard method for assessing multiple somatosensory submodalities. Experiment 1 showed that perception of warm temperature was the only somatosensory sub-modality that differed between HA and LA, with HA showing a higher threshold for detecting warm temperature than LA. Many QST batteries measure cold and warm thresholds in separate blocks of trials (Rolke, Baron et al., 2006, Rolke, Magerl et al., 2006). This practice could potentially confound sensitivity to thermal stimuli with response bias, so that between-group differences in thresholds could reflect differences in bias rather than in perceptual sensitivity. For example, a liberal decision criterion, due to some non-perceptual factor such as trait impulsivity, would lead to low thresholds, and could be mistaken for high thermosensitivity. Importantly, we randomly intermixed cold and warm stimuli, and asked participants to identify the direction of temperature change that they had detected. In this arrangement, a participant with a liberal decision criterion would make less accurate judgments than one with a more conservative criterion. Crucially, we found no difference in accuracy between HA and LA groups in detecting either warm or cold temperature. While this null result cannot rule out any contribution from response bias, it does clarify interpretation of our threshold measures. Specifically, the higher threshold for warmth detection in the HA group, compared to the LA group, appears to be a genuine difference in perceptual sensitivity within the warm thermoreceptive pathway, rather than merely a response bias.

Experiment 2 sought to replicate this result in a new sample, and across the entire distribution of alexithymia trait expression. We modelled thermoception thresholds as a continuous function of alexithymia scores, and found a strong linear relation between warmth perception threshold and level of alexithymia, with higher levels of alexithymia being associated with lower sensitivity for warmth. Exploratory post-hoc sub-analyses of different TAS subscales suggested the correlation was due to the feeling facets of alexithymia, rather than external orientation of thought. We found no other significant group differences. In particular, we found no effects of alexithymia on either affective touch, or on pain processing, in contrast to previous studies (Katz et al., 2009; Lumley, Smith, & Longo, 2002; Nyklíček & Vingerhoets, 2000).

Activation of the insula during emotion recognition is reduced in high alexithymics, possibly explaining their cognitive and affective impairments (Kano et al., 2003, Reker et al., 2010; see Moriguchi & Komaki, 2013 for a review). Interestingly, the insula also contributes to thermoception (Brooks, Nurmikko, Bimson, Singh, & Roberts, 2002; Craig, Chen, Bandy, & Reiman, 2000; Davis, Pope, Crawley, & Mikulis, 2004; Gelnar, Krauss, Sheehe, Szeverenyi, & Apkarian, 1999; Maihöfner, Kaltenhäuser, Neundörfer, & Lang, 2002; Moulton, Keaser, Gullapalli, & Greenspan, 2005; Sawamoto et al., 2000; for a review see Rolls, 2010). Activation in ventral posterior insula is correlated with the sensory properties of thermal stimuli. The insular cortex may function as a centre that relays information from sensory cortices to higher-order association cortex, influencing autonomic and visceral responses and consequently affects emotion recognition (Adolphs, 2002; Lang, Greenwald, Bradley, & Hamm, 1993). Therefore, alterations in insula activation in alexithymia might affect both perception of physical warmth, and social/emotional “warmth”. Thus, alexithimics reportedly lack emotional warmth and empathising with others while observing painful stimulation (Bird et al., 2010; Moriguchi et al., 2007).

Here we showed that in addition to cognitive and affective problems, alexithymia is associated with specific low-level somatosensory deficits, namely low sensitivity to warmth. Alexithymic deficits in cognitive processing of emotion might be linked to the functioning of low-level thermoceptive perceptual systems. Indeed, insensitivity to physical warmth could potentially explain the lack of social warmth found in high levels of alexithymia. In other words, cognitive and affective difficulties in alexithymia might be a consequence of defecate in low-level somatosensory processing, rather than a necessarily high-level cognitive deficit.

Unlike some previous studies, we used a comprehensive test battery, and submodality-selective tests to investigate the links between somatosensory function and alexithymia. The QST approach is inspired by the fact that different qualities of somatosensation are each transmitted by distinct neural pathways, associated with specific receptor types, and afferent fibre types. Thus, the specific association we found between alexithymia and warmth perception can be linked to neuroanatomical mechanisms. Importantly, we found effects that were defined by a neurophysiological afferent mechanism (warm), rather than by a physical stimulus dimensions or a perceptual dimension of temperature. That is, alexithymia was associated with altered perception of non-noxious warmth, yet perception of innocuous cold and of noxious heat were unaffected. The sensation of warmth is transmitted via unmyelinated C-fibres whereas nonpainful cold is conducted by small myelinated Aδ fibres (Schepers & Ringkamp, 2010). This may indicate that while the pathway for perception of cold is intact in HA, the warm-conducting pathways are specifically hypoactivated in persons with high alexithymia. Some neuroimaging studies suggest that the peripheral distinction between warm and cold processing is maintained in central thermoceptive processing. Both pathways project to the insula (Casey, Minoshima, Morrow, & Koeppe, 1996; Craig, Reiman, Evans, & Bushnell, 1996; Davis, Kwan, Crawley, & Mikulis, 1998; Olausson et al., 2005; Rolls, Grabenhorst, & Parris, 2008), but cold stimulation preferentially activated secondary somatosensory cortex (Casey et al., 1996), while warm stimulation mostly activates S1, anterior cingulate and the opercular-insular areas (Casey et al., 1996, Iannetti et al., 2003). This indicates that central processing of warmth and cold are at least partially dissociated.

Several studies have linked somatosensory processing to social-affective processing. For example, one very recent animal study suggested that a somatosensory deficit could underlie the development of autistic-like social and emotional behaviours (Orefice et al., 2016). In human cognitive neuroscience, areas such as insular cortex were implicated in processing both physical warmth but also social warmth (Inagaki & Eisenberger, 2013; Williams & Bargh, 2008).

Our study provides more mechanistic evidence for the possible missing link between sensation and social emotion in such arguments, at least in the case of alexithymic traits, by showing a plausible, low-level neural impairment relevant to alexithymia. The idea that physical and emotional warmth are linked is not new. Williams and Bargh (2008) reported that individuals with difficulty in emotion understanding and emotion expressing are less sensitive to physical warmth. Further, individuals with few warm social interactions reported taking more frequent hot showers. This was interpreted as an unconscious substitution of physical for social warmth (Bargh & Shalev, 2012). This effect remains controversial, and others failed to replicate the association (Donnellan, Lucas, & Cesario, 2015). Our study used the methods of quantitative neurophysiology, rather than those of social psychology, and targeted a very different research question. Nevertheless, our results potentially point to a mechanistic explanation for this general class of effect: we found that participants expressing the poor socio-affective cognition that characterises alexithymia also appeared relatively insensitive to warm stimulation.

Warm threshold was the only somatosensory function among the seven QST subtests of experiment 1 that was significantly linked to alexithymia scores. We cannot draw strong conclusions from those subtests that gave null results. However, the overall pattern across subtests does point to a specific link between alexithymic traits and the warm thermoceptive pathway. Importantly, the dissociation found in experiment 1 with warm, but not cold, thermoception was replicated in experiment 2 using a new, larger sample, covering a broader range of the population.

The results of one particular test deserve special comment. Several authors have speculated that the c-tactile mechanoreceptor pathway may play a special role in social emotion. This pathway’s unique role in pleasant touch might be relevant to behaviours such as grooming and caressing, providing a link between a specific somatosensory submodality and positive social emotion. We therefore included an affective touch test in our battery, although it is not a classic element of most QST batteries. Quantitative testing of affective touch is problematic, for several reasons. First, the relevant receptors and pathway cannot be activated selectively in healthy participants (but see Liljencrantz & Olausson, 2014; Olausson et al., 2002; ; Olausson et al., 2008): any stimulus that activates c-tactile mechanoreceptors will also activate light touch receptors and their associated Aβ fibres. Second, the assessment relies on the observation that subjective pleasantness of stroking varies with movement velocity with an inverted-U shaped tuning profile similar to the neural tuning curve of individual c-tactile afferents recorded microneurographically (Olausson et al., 2002). However, the similarity of neurographic and psychophysical tuning curves does not exclude the possibility that afferent pathways other than c-tactile, may also contribute to pleasantness. In our experiment 1, individuals with high alexithymia gave overall higher pleasantness ratings than those with low alexithymia, but no interaction with stroking velocity was found. In the absence of the predicted velocity-specific interaction, differences in overall ratings between the alexithymia groups might not reflect differences in specific sensory channels, but general biases in evaluation. Thus, we found no evidence for a *specific* deficit in affective touch pathways associated with alexithymia. Instead, we unexpectedly found that high alexithymics gave generally higher pleasantness ratings overall, but without any interaction with velocity.

Several limitations of our method and results should be kept in mind. Some QST subtests may have been too insensitive to detect differences between HA and LA. Thus, we may have missed associations between alexithymia and other sub-modalities, beyond warm thermoception. For instance, in interoceptive sensivity testing, participants’ body mass index, baseline measurement of ECG, and time perception abilities were not controlled. Recent studies (Herbert, Blechert, Hautzinger, Matthias, & Herbert, 2013; Meissner & Wittmann, 2011) suggest that these factors may also contribute to interoceptive sensitivity measurement. Second, our affective touch test differed from the other QST subtests in two ways. First, it was based on a subjective rating, rather than a classical psychophysical method. Second, it could not show the same level of pathway specificity as the other tests. Other limitations relate to the sample. We could not clinically assess all relevant comorbidities (e.g. anxiety, eating disorder, somatoform disorder, etc) although our online, self-report screening procedure did exclude severe depression, and history of any psychiatric or neurologic disorders.

Future studies needed to be done to further investigate the function of somatosensory pathways and whether the activation of somatosensory correlates differs during emotion processing in alexithymia. In particular, neuroimaging studies of brain responses to thermal stimuli in high and low alexithymic individuals could potentially yield neural evidence consistent with the lower sensitivity to warmth in high alexithymics.

Overall, the current study provides evidence that alexithymia does not involve only cognitive and affective deficits in emotion processing. Rather, it also involves low level somatosensory alterations, specific to the perception of warmth. The cognitive dimension of this trait might be partially grounded in the thermal somatosensory system.
