# Supplementary material for: “Lacking warmth”: Alexithymia trait is related to warm-specific thermal somatosensory processing
Source: Biol Psychol. 2017 Sep;128:132–40. doi: 10.1016/j.biopsycho.2017.07.012 (PMC5595273; doi:10.1016/j.biopsycho.2017.07.012)
Supplement: Supplementary file 1 [file mmc1.doc]

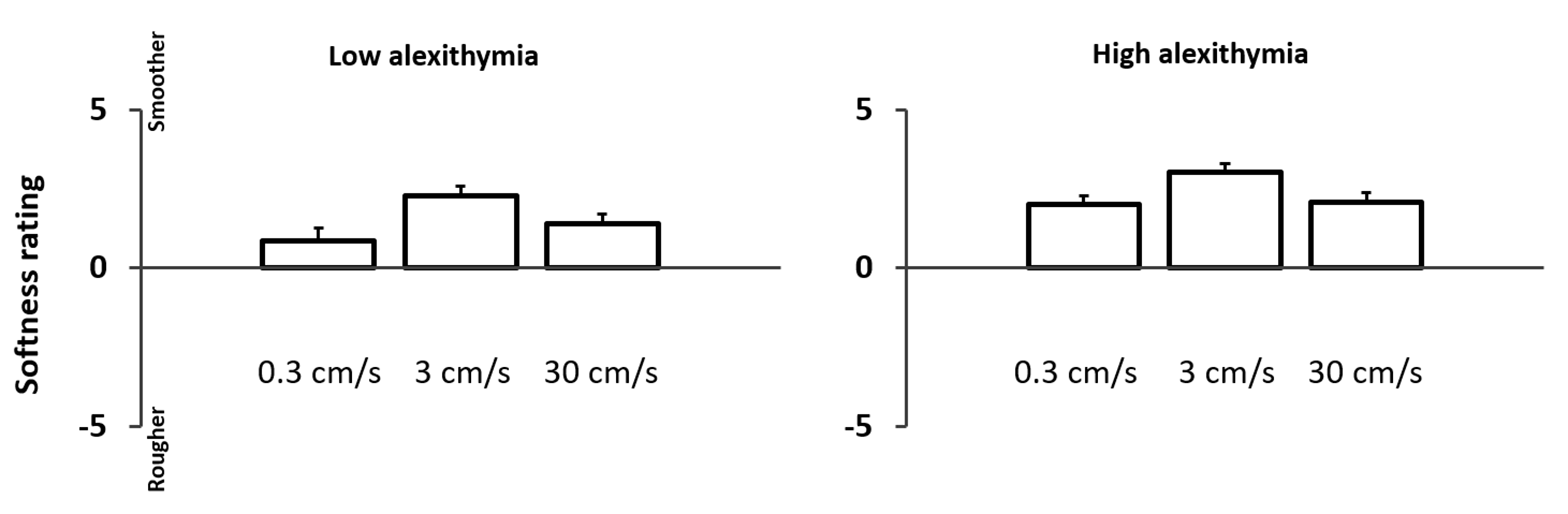


Supplementary figure S1. Mean softness ratings for the three different stroking velocities. Stroking at 3 cm/s was rated as significantly softer than stroking at 0.3 or 30 cm/s. Error bars show standard error across participants.
