# Supplementary material for: “Lacking warmth”: Alexithymia trait is related to warm-specific thermal somatosensory processing
Source: Biol Psychol. 2017 Sep;128:132–40. doi: 10.1016/j.biopsycho.2017.07.012 (PMC5595273; doi:10.1016/j.biopsycho.2017.07.012)
Supplement: Supplementary file 2 [file mmc2.doc]

**Supplementary material**

Supplementary table S1a

Means and standard deviations on the measure of pleasantness of affective touch in low and high alexithymia groups

| Pleasantness rating | | |  |
| --- | --- | --- | --- |
|  | | |  |
| Group *n* *M SD* | | |  |
| Low alexithymia | 20 0.92 1.64 | |  |
| High alexithymia | 20 1.87 1.21 | |  |
| Velocity |  | |  |
| 0.3 cm/s | 40 - 0.05 2.21 | |  |
| 3 cm/s | 40 2.52 1.57 | |  |
| 30 cm/s | 40 1.73 1.53 | |  |
| Agent |  | |  |
| Phantom | 40 1.30 1.60 | |  |
| Experimenter | 40 1.50 1.59 | |  |
|  | | |  |
| Interactions | | | |
| Robot 0.3 cm/s in low alexithymia | | 20 -0.60 2.69 | |
| Robot 3 cm/s in low alexithymia | | 20 1.72 1.98 | |
| Robot 30 cm/s in low alexithymia 20 1.50 1.75 | | | |
| Experimenter 0.3 cm/s in low alexithymia 20 -0.62 2.33 | | | |
| Experimenter 3 cm/s in low alexithymia 20 2.55 2.10 | | | |
| Experimenter 30 cm/s in low alexithymia 20 1.00 1.68 | | | |
| Robot 0.3 cm/s in high alexithymia 20 0.32 2.26 | | | |
| Robot 3 cm/s in high alexithymia 20 2.57 1.42 | | | |
| Robot 30 cm/s in high alexithymia 20 2.27 1.49 | | | |
| Experimenter 0.3 cm/s in high alexithymia 20 0.67 2.41 | | | |
| Experimenter 3 cm/s in high alexithymia 20 3.25 1.18 | | | |
| Experimenter 30 cm/s in high alexithymia 20 2.15 1.47 | | | |
| Robot 0.3 cm/s 40 -0.13 2.50 | | | |
| Robot 3 cm/s 40 2.15 1.76 | | | |
| Robot 30 cm/s 40 1.88 1.65 | | | |
| Experimenter 0.3 cm/s 40 0.02 2.43 | | | |
| Experimenter 3 cm/s 40 2.90 1.72 | | | |
| Experimenter 30 cm/s 40 1.57 1.67 | | | |
| Robot in low alexithymia 20 0.87 1.69 | | | |
| Experimenter in low alexithymia 20 0.97 1.74 | | | |
| Robot in high alexithymia 20 1.72 1.43 | | | |
| Experimenter in high alexithymia 20 2.02 1.24 | | | |
| 0.3 cm/s in low alexithymia 20 -0.61 2.29 | | | |
| 3 cm/s in low alexithymia 20 2.13 1.88 | | | |
| 30 cm/s in low alexithymia 20 1.25 1.58 | | | |
| 0.3 cm/s in high alexithymia 20 0.50 2.04 | | | |
| 3 cm/s in high alexithymia 20 2.91 1.09 | | | |
| 30 cm/s in high alexithymia 20 2.21 1.36 | | | |

Supplementary table S1b

Analysis of variance of pleasantness rating in affective touch

| Source *df* *SS* *MS* *F* *P* η_P_^2^ |
| --- |
| Group 38 54.15 54.15 4.30 0.04 0.10 |
| Velocity 76 279.68 139.84 46.39 0.00 0.54 |
| Agent 38 2.40 2.40 1.35 0.25 0.03 |
| Agent x Velocity X Group 76 0.91 0.45 0.35 0.69 0.00 |
| Agent x Velocity 76 11.33 5.66 4.43 0.01 0.10 |
| Agent x Group 38 0.60 0.60 0.33 0.56 0.00 |

Velocity x Group 76 1.14 0.57 0.19 0.82 0.00

Supplementary table S2a

Means and standard deviations on the measure of softness of affective touch in low and high alexithymia groups

| Softness rating | | |  |
| --- | --- | --- | --- |
|  | | |  |
| Group *n* *M SD* | | |  |
| Low alexithymia | 20 1.50 1.35 | |  |
| High alexithymia | 20 2.37 1.03 | |  |
| Velocity |  | |  |
| 0.3 cm/s | 40 1.43 1.84 | |  |
| 3 cm/s | 40 2.65 1.32 | |  |
| 30 cm/s | 40 1.73 1.45 | |  |
| Agent |  | |  |
| Phantom | 40 2.02 1.50 | |  |
| Experimenter | 40 1.85 1.36 | |  |
|  | | |  |
| Interactions | | | |
| Robot 0.3 cm/s in low alexithymia | | 20 0.90 2.51 | |
| Robot 3 cm/s in low alexithymia | | 20 2.12 1.90 | |
| Robot 30 cm/s in low alexithymia 20 1.65 1.60 | | | |
| Experimenter 0.3 cm/s in low alexithymia 20 0.80 2.60 | | | |
| Experimenter 3 cm/s in low alexithymia 20 2.40 1.38 | | | |
| Experimenter 30 cm/s in low alexithymia 20 1.12 1.65 | | | |
| Robot 0.3 cm/s in high alexithymia 20 2.50 1.78 | | | |
| Robot 3 cm/s in high alexithymia 20 2.75 1.39 | | | |
| Robot 30 cm/s in high alexithymia 20 2.20 1.60 | | | |
| Experimenter 0.3 cm/s in high alexithymia 20 1.52 1.68 | | | |
| Experimenter 3 cm/s in high alexithymia 20 3.32 1.32 | | | |
| Experimenter 30 cm/s in high alexithymia 20 1.97 1.46 | | | |
| Robot 0.3 cm/s 40 1.07 2.30 | | | |
| Robot 3 cm/s 40 2.43 1.67 | | | |
| Robot 30 cm/s 40 1.92 1.61 | | | |
| Experimenter 0.3 cm/s 40 1.16 2.19 | | | |
| Experimenter 3 cm/s 40 2.86 1.41 | | | |
| Experimenter 30 cm/s 40 1.55 1.60 | | | |
| Robot in low alexithymia 20 1.55 1.58 | | | |
| Experimenter in low alexithymia 20 1.44 1.44 | | | |
| Robot in high alexithymia 20 2.48 1.30 | | | |
| Experimenter in high alexithymia 20 2.27 1.18 | | | |
| 0.3 cm/s in low alexithymia 20 0.85 2.15 | | | |
| 3 cm/s in low alexithymia 20 2.26 1.42 | | | |
| 30 cm/s in low alexithymia 20 1.38 1.47 | | | |
| 0.3 cm/s in high alexithymia 20 2.01 1.26 | | | |
| 3 cm/s in high alexithymia 20 3.03 1.13 | | | |
| 30 cm/s in high alexithymia 20 2.08 1.36 | | | |

Supplementary table S2b

Analysis of variance of softness rating in affective touch

| Source *df* *SS* *MS* *F* *P* η_P_^2^ |
| --- |
| Group 38 46.37 46.37 5.32 0.02 0.12 |
| Velocity 76 64.31 32.15 13.03 <0.0001 0.25 |
| Agent 38 1.58 1.58 0.55 0.45 0.03 |
| Agent x Velocity X Group 76 4.60 2.30 1.67 0.19 0.04 |
| Agent x Velocity 76 10.61 5.30 3.87 0.02 0.09 |
| Agent x Group 38 0.12 0.12 0.04 0.83 0.00 |

Velocity x Group 76 2.46 1.23 0.49 0.60 0.01
